# The Integration of Clinical Decision Support Systems Into Telemedicine for Patients With Multimorbidity in Primary Care Settings: Scoping Review

**DOI:** 10.2196/45944

**Published:** 2023-06-28

**Authors:** Nutchar Wiwatkunupakarn, Chanchanok Aramrat, Suphawita Pliannuom, Nida Buawangpong, Kanokporn Pinyopornpanish, Nopakoon Nantsupawat, Poppy Alice Carson Mallinson, Sanjay Kinra, Chaisiri Angkurawaranon

**Affiliations:** 1 Department of Family Medicine Faculty of Medicine Chiang Mai University Chiang Mai Thailand; 2 Global Health and Chronic Conditions Research Group Chiang Mai University Chiang Mai Thailand; 3 Department of Non-communicable Disease Epidemiology Faculty of Epidemiology and Population Health London School of Hygiene and Tropical Medicine London United Kingdom

**Keywords:** telemedicine, clinical decision support system, CDSS, primary care, multimorbidity, polypharmacy, chronic disease, pharmacy, pharmaceutic, telehealth, decision support, scoping, search strategy, review

## Abstract

**Background:**

Multimorbidity, the presence of more than one condition in a single individual, is a global health issue in primary care. Multimorbid patients tend to have a poor quality of life and suffer from a complicated care process. Clinical decision support systems (CDSSs) and telemedicine are the common information and communication technologies that have been used to reduce the complexity of patient management. However, each element of telemedicine and CDSSs is often examined separately and with great variability. Telemedicine has been used for simple patient education as well as more complex consultations and case management. For CDSSs, there is variability in data inputs, intended users, and outputs. Thus, there are several gaps in knowledge about how to integrate CDSSs into telemedicine and to what extent these integrated technological interventions can help improve patient outcomes for those with multimorbidity.

**Objective:**

Our aims were to (1) broadly review system designs for CDSSs that have been integrated into each function of telemedicine for multimorbid patients in primary care, (2) summarize the effectiveness of the interventions, and (3) identify gaps in the literature.

**Methods:**

An online search for literature was conducted up to November 2021 on PubMed, Embase, CINAHL, and Cochrane. Searching from the reference lists was done to find additional potential studies. The eligibility criterion was that the study focused on the use of CDSSs in telemedicine for patients with multimorbidity in primary care. The system design for the CDSS was extracted based on its software and hardware, source of input, input, tasks, output, and users. Each component was grouped by telemedicine functions: telemonitoring, teleconsultation, tele–case management, and tele-education.

**Results:**

Seven experimental studies were included in this review: 3 randomized controlled trials (RCTs) and 4 non-RCTs. The interventions were designed to manage patients with diabetes mellitus, hypertension, polypharmacy, and gestational diabetes mellitus. CDSSs can be used for various telemedicine functions: telemonitoring (eg, feedback), teleconsultation (eg, guideline suggestions, advisory material provisions, and responses to simple queries), tele–case management (eg, sharing information across facilities and teams), and tele-education (eg, patient self-management). However, the structure of CDSSs, such as data input, tasks, output, and intended users or decision-makers, varied. With limited studies examining varying clinical outcomes, there was inconsistent evidence of the clinical effectiveness of the interventions.

**Conclusions:**

Telemedicine and CDSSs have a role in supporting patients with multimorbidity. CDSSs can likely be integrated into telehealth services to improve the quality and accessibility of care. However, issues surrounding such interventions need to be further explored. These issues include expanding the spectrum of medical conditions examined; examining tasks of CDSSs, particularly for screening and diagnosis of multiple conditions; and exploring the role of the patient as the direct user of the CDSS.

## Introduction

Multimorbidity, the presence of more than one condition in a single individual [1], is a global phenomenon that has impacted individual health and imposed national economic burdens [2-5]. People with multiple illnesses tend to have a lower quality of life, higher disability, and higher incidence of coexisting acute diseases compared to those without multiple illnesses [6,7]. It has been found that multimorbidity increases health and hospital expenditures through increased costs for medication, outpatient care, and hospitalization [2-5,8]. The raised workload on the medical staff, especially in a primary care setting, results in a lower quality of care. This results in patients with multimorbidity becoming less likely to receive patient-centered care and appropriate continuity of care [9,10].

Providing effective care for people with multimorbidity is complex, as patients and health providers face many challenges [11-15]. From the patient’s perspective, one of the barriers to reaching effective health outcomes is the complexity of the care process, which includes multiple appointments, refilling multiple prescriptions, and difficulty in understanding complex medical details. Patients are burdened by this process, particularly when all disease guidelines are followed [14]. Another barrier is the difficulty of access to care. As medical resources are scarce, the provision of health care facilities might be insufficient, leading to inadequate service centers, consultation restrictions, and limited geographic access [11-13]. From a provider’s perspective, multimorbidity contributes to more complex disease management and increased workload [15]. Patients with multiple diseases are also usually affected by polypharmacy, requiring more awareness of drug interactions and adverse events [16,17].

Information and communication technology (ICT) has been introduced to help deal with the complexities and challenges of caring for patients with multimorbidity [18]. Common ICT in health care services includes telemedicine and clinical decision support systems (CDSSs). Telemedicine, or telehealth, refers to the use of technology to improve patient outcomes by delivering health service at a distance. It comprises several basic medical care functions, including monitoring, consulting, mentoring, and providing medical knowledge [19]. Telemedicine has been claimed as an essential tool for improving health service access (increasing convenience of health care visits and removing geographic barriers) and reducing patient burdens from complex care processes (decreasing the frequency of appointments, providing necessary medical knowledge, and enhancing patient self-management) [19-23]. CDSSs are another common ICT used in health care. They have been developed in an attempt to assist health providers in making medical decisions by reducing the complexity of disease management. Computer-based programming has been used to analyze patient data from electronic health records (EHRs) and integrate the analyzed results with clinical knowledge and guidelines. The program then offers suitable choices for patients and providers through prompts, reminders, or treatment recommendations at the appropriate times [24]. These decision-support tools can be helpful for providers when managing patients with multiple conditions, as they reduce the burden and complexity of analyzing multiple problems and integrating different clinical guidelines. Assisting providers in complex decision-making allows patients to obtain better clinical outcomes and promotes both patient and provider satisfaction [25].

Both telemedicine and CDSSs could be potential tools for strengthening the capability and capacity of health care services in caring for multimorbidity in primary care settings where medical resources are limited [18]. However, each element of telemedicine and CDSSs is often examined separately as a tool for caring for multimorbidity, and there is great heterogeneity in implementation. Telemedicine has been used for simple patient education as well as more complex consultations and case management. CDSSs have variability in data inputs, intended users, and actions (ie, outputs). Thus, there are several gaps in knowledge about how to integrate CDSSs into telemedicine. Moreover, it is uncertain to what extent these integrated technological interventions can help improve outcomes for patients with multimorbidity. Therefore, the aims of this review are to (1) provide a broad review of how CDSSs have been integrated into each function of telemedicine for multimorbid patients in primary care, (2) summarize the effectiveness of telemedicine and CDSS interventions by looking at clinical and patient outcomes, and (3) identify gaps in the literature.

## Methods

This scoping review used the PRISMA (Preferred Reporting Items for Scoping Reviews) checklist. The review protocol was registered on PROSPERO (CRD42021293444). Scoping reviews help identify how research is conducted in a given area, identify key characteristics related to particular concepts, and identify and analyze research gaps [26].

### Search Strategy

An online literature search was conducted in September 2021 and updated in November 2021. The electronic databases included PubMed, Embase, CINAHL, and Cochrane. Only English articles published from 2015 to 2021 were included. We limited the publications to those published after 2015 due to the fact that CDSSs only started to be commonly used in health care, health science, and clinical informatics in 2015 [27]. Online databases showed a significant increase in the number of publications related to the use of CDSSs in telemedicine in 2015 and an exponential increase since the COVID-19 pandemic [28]. Additional checking of the reference lists of relevant papers was also done. The initial search terms were based on 2 concepts: *clinical decision support system* AND *multimorbidity* (eg, multiple conditions, multidrug therapy, concurrent illness, polypharmacy). The term *telemedicine* was considered in the articles that had both search terms. The full search terms are shown in Multimedia Appendix 1. An additional reference search was performed from articles included in the review. An extended search was then performed by adding more search terms for several specific conditions commonly examined in multimorbidity found in the initial searches. These conditions included diabetes, cardiovascular disease, hypertension, dyslipidemia, and heart failure. Additional search terms are shown in Multimedia Appendix 2. In this review, aside from having 2 or more conditions in a single individual, the word *polypharmacy* (ie, the act of taking ≥5 medicines) was inferred as implying multimorbidity, since prescribing 5 or more drugs for only 1 disease is rare [29].

### Eligibility Criteria

The inclusion criteria were (1) the study focused on patients with multiple chronic conditions; (2) the intervention included any services using telemedicine and a CDSS; and (3) the setting was restricted to primary care.

We excluded (1) studies investigating telemedicine alone or a CDSS alone; (2) studies including patients with a single disease or multiple diseases that were the complication or consequence of each other; (3) studies without reported results, such as protocols, editorials, comments, perspectives, and correspondence; and (4) review articles.

### Study Selection

Once duplicate results were removed, 4 authors (NW, SP, NB, and KP) were responsible for the screening and data extraction process. Abstract and full-paper screening was performed by at least 2 authors independently for each paper. Any disagreement on eligibility was identified and resolved by the senior author (C Angkurawaranon) if necessary.

### Data Extraction

**Figure 1:**
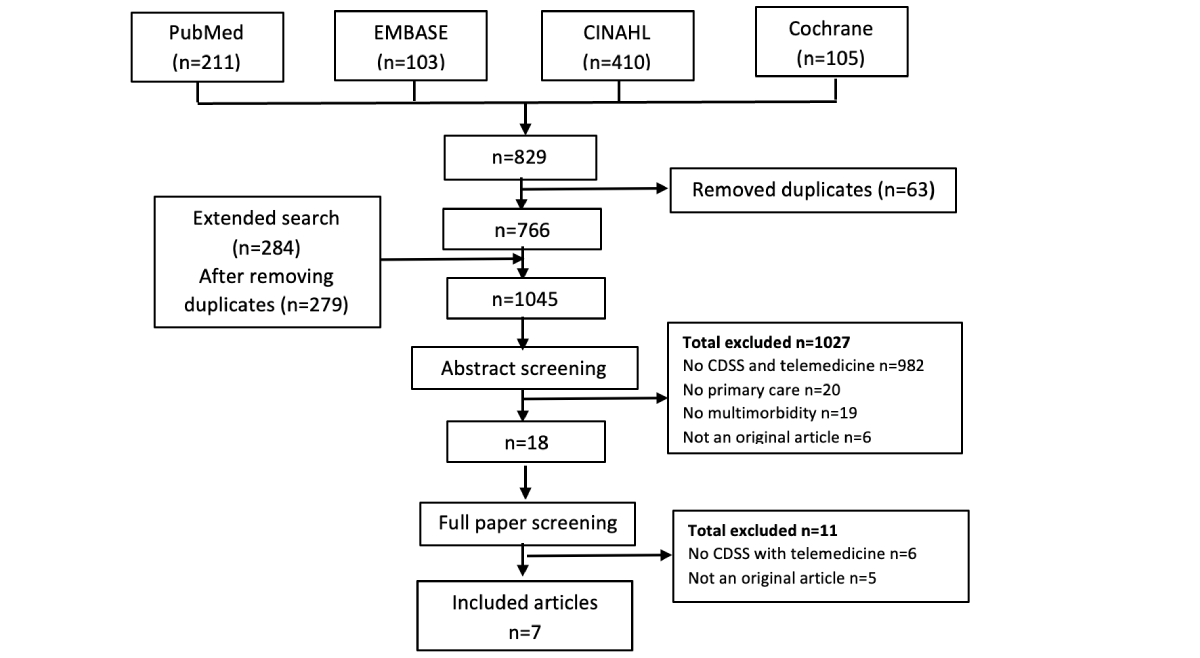
Flow chart of the selection process in different databases. CDSS: clinical decision support system.

Similar to the screening process, 4 authors (NW, SP, NB, and KP) were responsible for data extraction. For each study selected, data were extracted by 2 authors working separately, and any disagreement was discussed with the senior author. The data included the author’s name, year of publication, year the study was conducted, country, study design, population characteristics, mean age of the population, sample size, details of the intervention, and outcomes of the studies in all methods of measurement (eg, mean difference, risk ratio, and odds ratio). Data from the same trial but with results published as multiple papers are summarized as 1 trial. The flow chart of the selection process is illustrated in Figure 1.

### Data Items

#### Telemedicine and CDSSs

We extracted data on telemedicine components and the structure of the CDSSs by adopting the definition of telemedicine components of Lee et al [19] and the structure of CDSSs from Fraccaro et al [30]. The structure of the CDSSs was extracted into 6 domains: software and hardware, source of input, input data, task, output (ie, content), and users and decision makers. The tasks of the CDSSs were categorized into 6 main purposes: disease prevention; disease diagnosis; patient management according to guidelines (or pathways); medication-related issues, such as drug interactions; patient education; and patient self-management.

For telemedicine, we used a classification modified from Lee et al [19]. We classified telemedicine into four categories: (1) tele-mentoring or teleconsultation, (2) tele–case management, (3) telemonitoring, and (4) tele-education. Tele-mentoring, which includes teleconsultation, is defined as the provision of medical guidance, disease-specific suggestions, and specific answers to patients by health providers. Tele–case management is defined as any communication between primary care providers and other specialists, case transferal, case referral, or medicine adjustment. The definitions of telemonitoring and tele-education followed the original taxonomy by Lee et al [19], defined as the process of distant transmission of patients’ health status and the use of technologies for the delivery of distance learning, respectively.

#### Effectiveness of Telemedicine and CDSSs

The effectiveness of the interventions was initially determined by looking at clinical outcomes or indicators of disease control, including biomarkers, such as hemoglobin A_1C_ (HbA_1c_), fasting blood sugar level, blood pressure, body weight, and BMI.

Other outcomes of interest included medication management, patient self-management, and disease management. Medication management included documented adverse drug events (ADEs), medication errors, and medication discontinuation. Patient self-management included compliance with self-monitoring (such as home monitoring of blood pressure and capillary blood sugar level) and change in health behaviors. Finally, disease management was assessed by the ability of the system to provide diagnoses and further steps according to practice guidelines at the appropriate time. Other outcomes beyond this scope but related to the objectives were also included if reported.

### Risk of Bias Assessment

As no observational studies were included, the Cochrane Risk of Bias Tool (version 2) was used to assess the risk of bias among experimental studies as low, high, or unclear [31]. The biases assessed had five aspects: (1) bias arising from the randomization process, (2) bias due to deviations from intended interventions, (3) bias due to missing outcome data, (4) bias in the measurement of the outcome, and (5) bias in the selection of the reported results. This step was performed by 2 authors independently, and any disagreement between them was resolved by discussion with the senior authors.

### Synthesis of Results

For each study, the extracted data were qualitatively summarized. Key characteristics across studies were summarized using counts and percentages. The extracted data showed heterogeneity in study populations, designs, interventions, and outcomes. Therefore, meta-analysis of outcomes was not conducted.

## Results

### Search Selection

There were 829 potentially relevant publications from 4 online databases. Abstract screening for eligibility was done by 2 authors independently, and 25 full-text articles were assessed. Finally, 7 articles were included and extracted in this review. The flow chart of the selection process is illustrated in Figure 1.

### Characteristics of Included Studies

All articles included in this review were experimental studies published between 2017 and 2020: 3 RCTs [32-34] and 4 non-RCTs [35-38]. They were mostly conducted in high-income countries such as the United States [32,33], England [38], Canada, Italy, and Spain [37]. Participants were patients with ≥2 chronic conditions aged from 33 years to older than 60 years. The existing chronic conditions included in this review were diabetes mellitus [32,34,35,38], hypertension [31,33,34,36,37], atrial fibrillation [37,38], and polypharmacy [33,36], with only 1 study focusing on diabetes mellitus in pregnancy [37]. The details of each study by year and chronic conditions are shown in Table 1.

**Table 1:** Characteristics of included studies.

Author (country), year of publication	Year conducted, design	Sample size (n), participant characteristics	Conditions	Age (years), mean	Intervention	Follow-up (months)	Control	Outcome
Fried [32] (US), 2017	2014-2016, RCT^a^	128 community-dwelling veterans aged ≥65 years	HT^b^, DM^c^, polypharmacy	N/A^d^	TRIM^e^ app	3	TRIM telephone assessment plus usual care or usual care alone	Patient involvement; clinician-patient communication; correction of medication discrepancies; reduction in number of medications
Marcolino [35] (Brazil), 2021	2016-2018, quasi-experimental study	4211 patients registered by primary care team, 25 physicians, 44 nurses, 27 other professionals	HT, DM	N/A for patients; median 33 for others	CDSS^f^	6	None	User satisfaction
McDonald [36] (Canada), 2019	2016-2017, experimental study using cohort as a control	800 patients aged ≥65 years admitted to an IPD^g^ and covered by health insurance and a prognosis of more than 3 months	Polypharmacy	Intervention: median 81; control 79	MedSafer	1	Usual care: BPMH^h^ with medication reconciliation at admission and discharge (no education or telemedicine)	Number of patients who had documented potentially inappropriate medications; adverse drug events within 30 days of hospital discharge
Peleg [37] (Italy, Spain), 2017	2010-2015, experimental study using cohort as a control	266 pregnant woman, aged >35 years with AF^i^	Gestational diabetes mellitus with/without HT, AF	Intervention: 35.2; control: 34	MobiGuide	4	Historical gestational diabetes mellitus cohort	Compliance to blood glucose, urine ketone, and blood pressure measurements; number of clinicians starting insulin therapy; compliance to electrocardiogram and blood pressure measurements; number of changes in diagnosis and treatment
Schiff [33] (US), 2019	2013-2015, RCT	1152 patients aged >18 who were English or Spanish speakers	Polypharmacy	Intervention 57.2; control 59.7	IVR^j^ script and pharmacist protocol	12	Usual care	Adverse effect of medication; medication discontinuation with reasons
Willis [38] (England), 2020	2015-2016, RCT	178 general practioners covering 2.2 million residents	DM, HT, AF, risky prescription	38.4	SystmOne	11	No intervention	DM control; risky prescribing; blood tests; pressure control; anticoagulation in AF
Prabhakaran [34] (India), 2018	2016-2017, RCT	3324 patients aged ≥30 years intending to reside in the catchment area of the CHC^k^ for ≥1 year	Uncontrolled HT or uncontrolled DM	Intervention 56.7; control 55.5	mWellcare system	12	Usual care (clinical management guidelines for HT and DM)	Change in systolic blood pressure, hemoglobin A_1C_, free blood sugars, total cholesterol, cardiovascular disease risk, tobacco use, BMI, alcohol use, and depression score

^a^RCT: randomized controlled trial.

^b^HT: hypertension.

^c^DM: diabetes mellitus.

^d^N/A: not available.

^e^TRIM: Tool to Reduce Inappropriate Medications.

^f^CDSS: clinical decision support system.

^g^IPD: inpatient department.

^h^BPMH: best possible medication history.

^i^AF: atrial fibrillation.

^j^IVR: interactive voice response.

^k^CHC: community health center.

### Risk of Bias in Studies

In general, most studies were considered as having a low risk of bias for missing outcome data, measurement bias, and reporting bias. However, a high risk of bias due to a lack of randomization was found in several articles [35-37], and unclear evidence of performing an intention-to-treat analysis was found in 5 studies [33-38]. The results of the risk of bias assessment are illustrated in Table 2.

**Table 2:** Risk of bias summary.

	Bias arising from the randomization process	Bias due to deviations from intended interventions	Bias due to missing outcome data	Bias in measurement of the outcome	Bias in selection of the reported results
Fried et al [32] 2017	No	No	No	No	No
McDonald et al [36] 2019	Yes	Uncertain	No	No	No
Peleg et al [37] 2017	Yes	Uncertain	No	No	No
Schiff et al [33] 2018	No	Uncertain	No	Uncertain	No
Willis et al [38] 2020	No	Uncertain	No	No	No
Prabhakaran et al [34] 2018	No	No	No	No	No

### System Design of CDSSs in Telemedicine

#### Overview

Among the 7 included papers, telemedicine interventions were mainly used for monitoring, consulting, and case management (Multimedia Appendix 3 [32-38]). Only 1 study mentioned tele-education. This review found that the functions of telemedicine in treatment for multimorbidity often overlapped and that there often was more than 1 function in a single intervention, especially for tele–case management, which was not used alone without combination with other functions, such as telemonitoring. The tasks of CDSSs in telemedicine can be summarized into 6 categories. They were predominantly used for patient management according to guidelines, medication-related issues, disease diagnosis, patient education, and patient self-management, but not for disease prevention (Multimedia Appendix 4 [32-38]). This review found that CDSSs can complete several tasks, that is, they can manage multiple responsibilities at once, as demonstrated by their ability to provide notifications to patients and recommendations to providers at the same time. The following sections provide an overview of the system designs of CDSSs according to their main functions in telemedicine.

#### CDSS Designs for Use in Telemonitoring

Three papers reported interventions that used telemedicine for remote monitoring of patients with polypharmacy, atrial fibrillation, and gestational diabetes mellitus, as well as patients who were taking medicine to treat hypertension, diabetes, insomnia, and depression [32,33,37]. Sources of data input for the CDSSs varied, including manual assessment over the phone, wearable sensors, self-entering in a smartphone app, and EHRs. These sources gathered the necessary information from patients into the CDSS, which then detected ADEs, interpreted the data from sensors, and mapped patient data to clinical guidelines. The type of output depended on the task (Table 3). For example, CDSSs used for ADE detection and sensor interpretation may give feedback in the form of drug alerts and clinical issue notifications, respectively. In contrast, CDSSs used for guideline and health record interpretation may show outputs in the form of recommendations for patient management. Feedback was mostly sent to health providers, who were the main decision-makers in the patient care process. However, in one intervention, feedback was directly sent to patients [37], because they are the ones who decide their further management, such as visiting the outpatient department and modifying their behavior.

**Table 3:** Overview of system designs used by clinical decision support systems for telemonitoring.

Study (intervention), patient characteristics	Clinical decision support system							Outcomes of intervention
	Software/hardware	Source of input	Input	Task	Output (content)	Output users (ie, decision-makers)		
Fried et al [32] (TRIM^a^), men aged ≥65 years with polypharmacy (diabetes mellitus, hypertension, and others)	Web application/ computer	EHRs^b^; patient telephone assessment	Patient cognition, social support, potential overtreatment, renal dosing, patient reports of adverse drug events	Evaluate medical appropriateness; generate algorithms for medication management	Patient-specific medication management (complete medication reconciliation, recommendations for discontinuation or dosage changes, recommendation of simplified regimen for patient with poor drug compliance)	Clinicians	Higher patient involvement rate; more active patient communication; more facilitative clinician communication; more medication-related communication; more correction of medication discrepancies; no reduction of medication number	
Peleg et al [37] (MobiGuide), atrial fibrillation patients	App/smart phone	Personal health records (EHRs, sensors, patient-reported symptoms, data)	Patient status, symptoms, ECG^c^ measurement, blood pressure, international normalized ratio (taking warfarin), weight and exercise	Analyze data from ECG; map computer-interpretable guidelines to health record data	Patient recommendation, notification for patients (ECG, medications), notification for provider	Patients, clinicians	Higher compliance to ECG measurements and blood pressure measurements; more clinicians change their diagnosis and treatment	
Peleg et al [37] (MobiGuide), women aged 30-40 years with gestational diabetes mellitus (with or without hypertension)	App/smart phone	Personal health records (EHRs, sensors, patient-reported symptoms)	Patient status, symptoms, blood pressure, blood glucose level, ketonuria, exercise	Analyze data; map computer-interpretable guidelines to health record data	Patient recommendation, notification for patients, notification for provider	Patients, clinicians	Higher compliance to blood glucose levels, ketonuria, blood pressure; more clinicians start insulin therapy earlier; small number of patients received insulin therapy earlier	
Schiff et al [33], patients aged >18 years who newly received target medication with >7 doses	Interactive voice response/ telephone	EHRs, patient interviews	Patient status; number of primary care visits; target drug; history of diabetes mellitus, hypertension, depression, and insomnia; target medication adherence; drug-specific symptoms	Detect a positive symptom of adverse drug events	Adverse drug event alert	Pharmacists	More adverse drug event documentation in medical record; slightly more medication discontinuation with reasons	

^a^TRIM: Tool to Reduce Inappropriate Medications.

^b^EHR: electronic health record.

^c^ECG: electrocardiogram.

#### CDSS Designs for Use in Teleconsultation

Four studies investigated the use of telemedicine for consultation [34,35,37,38]. Health conditions examined included diabetes, hypertension, atrial fibrillation, and polypharmacy. CDSSs were used to confirm diagnoses based on patients’ information and diagnostic criteria, generate treatment plans based on the guidelines, and provide appropriate advisory materials for those with risky behaviors (Table 4). Some simple feedback created by the system was sent back to patients, including reminders for follow-up visits and medication adherence, general advice for patient self-care, and automated answers to patients’ common queries. However, for more complicated management and responses to more complex queries, health providers were offered treatment choices, and it was their task to answer patients’ questions using the information given by the CDSS.

**Table 4:** Overview of system design of clinical decision support systems for teleconsultation.

Study (intervention), patient characteristics	Clinical decision support system							Outcomes of intervention
	Software/hardware	Source of input	Input	Task	Output (content)	Output users (decision-makers shown in italics)		
Peleg et al [37] (MobiGuide); atrial fibrillation patients	App/smart phone	Personal health records (EHRs^a^, sensors, patient-reported symptoms and data)	Patient status, symptoms, ECG^b^ measurement, blood pressure, international normalized ratio (taking warfarin), weight and exercise	Analyze data from ECG; map computer-interpretable guidelines to health record data	Patient recommendations, notifications for patients (ECG, medications), notifications for providers	*Patients, clinicians*	Higher compliance to ECG and blood pressure measurements; more clinicians changed their diagnosis and treatment	
Peleg et al [37] (MobiGuide), women aged 30-40 years with gestational diabetes mellitus with or without hypertension	App/smart phone	Personal health records (EHRs, sensors, patient-reported symptoms)	Patient status, symptoms, blood pressure, blood glucose level, ketonuria, exercise	Analyze data; map computer-interpretable guidelines to health record data	Patient recommendations, notifications for patients, notifications for providers	*Patients, clinicians*	Higher compliance to blood glucose, urine ketone, and blood pressure; small number of patients received insulin therapy earlier	
Prabhakaran et al [34] (mWellcare), Patients aged ≥30 years with hypertension, diabetes mellitus, comorbid depression, and alcohol and tobacco use	Android app/ tablet computer	EHRs	Patient profile, diagnosed condition, comorbid conditions, previous and current medications	Generate treatment plan based on patient’s data and clinical guidelines	Recommended treatment plan, lifestyle modification advisory, date for the next follow-up visit, reminders for follow-up visit and medication adherence	Patients, *clinicians*	No improvement of biomedical parameters, no difference in tobacco or alcohol use	
Marcolino et al [35], patients with hypertension and diabetes mellitus	Application/computer	Data registry from health care professionals	Symptoms, medical history, physical examination, current medications, laboratory results and other complementary examinations	Screen for disease; confirm diagnosis; suggest clinical recommendations	Alerts and reminders of important clinical issues; recommendations for clinical management	Patients, *health care professionals*	All users were satisfied with the program	
Willis et al [37] (SystmOne), patients with hypertension, diabetes mellitus, atrial fibrillation, or risky prescription	Unknown/computer	EHRs, paper-based health records	Patient status, diagnosis, drug, duration of medication	Behavioral change technique delivery via audit and feedback and prompts and reminders	Audit and feedback (evidence-based clinical messages, answers to common queries, action-planning templates) and prompts and reminders (prompts for risky prescribing, prompts for adherence of quality-of-care indicators)	Patients, *clinicians*	The program has good performance for simple behaviors within the control of clinicians, but not for the behaviors that need patient engagement	

^a^EHR: electronic health record.

^b^ECG: electrocardiogram.

#### CDSS Designs for Use in Tele–Case Management

Two articles evaluated the use of CDSSs in managing patients with diabetes, hypertension, and polypharmacy [35,36]. Input data was mostly entered by health care providers from the patient registry and admission notes, including information regarding chief concerns, current illness, past medical history, current medications, laboratory results, and imaging. The outputs were notifications, clinical management recommendations, and medication management (Table 5). However, feedback was used among primary care providers and patients and could also be used between primary care providers and other specialists. For example, in the study by McDonald et al [36], patients’ prescriptions were circulated between community health providers and tertiary health facilities. Moreover, ICT systems were also designed for multiple-user registration, which increases the accessibility of systems to serve multidisciplinary teams, including physicians, nurses, nurse technicians, and support teams.

**Table 5:** Overview of system design of clinical decision support systems for tele–case management and tele-education.

Study (intervention), patient characteristics			Clinical decision support system													Outcomes of intervention
			Software/hardware		Source of input		Input		Task		Output (content)		Output users (decision-makers shown in italics)			
**Tele–case management**																
	McDonald 2019 et al [36] (MedSafer), patients aged ≥65 years with polypharmacy			Admission notes, EHRs^a^		Clinic visits, past hospitalization, past medical history, disease conditions		Identify deprescribing rules; screen for prescriptions; choose medication lists		Medication tapering instructions, patient education materials		Patients, caregivers, community pharmacists, *community physicians*		Increased documentation of patients with potentially inappropriate medications, slightly fewer ADEs^b^		
	Marcolino et al [35], patients with HT and DM	Application/computer		Data registry from health care professionals		Symptoms, medical history, physical examination, current medications, laboratory results and other complementary examinations		Screen for disease; confirm diagnosis; suggest clinical recommendation		Alerts and reminders of important clinical issues, recommendations for clinical management		Patients, *health care professionals*		All users were satisfied with the program		
**Tele-education**																
	McDonald et al [36] 2019 (MedSafer), patients aged ≥65 years with polypharmacy	N/A^c^		Admission notes, EHRs		Clinic visits, past hospitalization, past medical history, disease conditions		Identify deprescribing rules; screen for prescriptions; choose medication lists		Medication tapering instructions, patient education materials		Patients, caregivers, community pharmacists, *community physicians*		Increased documentation of patients with potentially inappropriate medications, slightly fewer ADEs		

^a^EHR: electronic health record.

^b^ADE: adverse drug event.

^c^N/A: not available.

#### CDSS Designs for Use in Tele-Education

Only one study investigated the use of CDSSs for tele-education [36]. This system was used for patients with polypharmacy to interpret the patients’ medication data according to deprescribing rules and screening for high-risk prescriptions (Table 5). The system instructed patients with medication-related educational content and provided suggestions on tapering to providers. Patients or caregivers spontaneously received learning materials related to their current medications; for example, they learned details on the harms of polypharmacy and on deprescription.

### Effectiveness of the Interventions and Other Outcomes

Six different types of outcomes were assessed in the 7 studies, including disease control, medication management, patient self-management, patient care, doctor-patient communication, and feasibility and satisfaction of intervention (Tables 2-5 and Multimedia Appendix 5 [32-38]). For disease control, 1 study conducted with patients with hypertension or diabetes showed that biomedical parameters were not significantly different from baseline to after completion of the intervention [34]. These parameters included blood pressure, BMI, fasting blood glucose level, HbA_1c_, and lipid profile. Three studies reported positive outcomes for medication management among the intervention groups, with more correction of medication discrepancies, fewer ADEs, and more appropriate medication discontinuation. However, the number of medications did not significantly decrease [32,33,36]. For patient self-management, 3 studies reported related outcomes [34,37,38]. Participants in the intervention group had higher compliance to biomarker monitoring (ie, electrocardiograms, blood glucose, blood pressure, and urine tests). Still, a significant change in risky behaviors was not seen (eg, tobacco and alcohol use). For disease management, the interventions improved the accuracy of diagnoses and shortened the time from diagnosis to initiation of treatment [37]. Clinicians who use CDSSs as smartphone apps were more likely to change their diagnoses and treatments to more appropriate ones; for example, they were more likely to start early insulin therapy for patients with diabetes.

Other associated outcomes assessed were communication between providers and patients and the program’s feasibility. For doctor-patient communication, the interventions enhanced patient participation, as can be seen by a higher patient involvement rate, more active patient communication, more facilitative clinician communication, and more medication-related communication [32]. Users from multidisciplinary teams that included physicians, nurses, and other health professionals demonstrated satisfaction with the intervention [35], but no satisfaction assessment was performed among patients.

## Discussion

### Principal Findings

This scoping review aims to explore how CDSSs can be integrated into telemedicine services for caring for people with multimorbidity. Our review found that CDSSs have been incorporated for telemonitoring, teleconsultation, tele–case management, and tele-education. However, the structure of CDSSs, such as data input, tasks, output, and intended users or decision-makers, varied. With limited studies, mostly from developed countries, and inconclusive evidence on the clinical effectiveness of the interventions, more research is needed to examine how CDSSs can be successfully integrated to improve patient outcomes in multimorbidity.

The evidence suggested that CDSSs can be integrated into telemedicine and used to improve the capacity of telehealth services. Telemonitoring is commonly used for multimorbidity, as it allows patients to update their health-related information (eg, blood tests and health behaviors) via online platforms and monitor patient health status (eg, blood pressure and electrocradiograms) in real-time by connecting with remote sensors or wearable devices. CDSSs play an important role in detecting abnormalities and giving alerts or feedback to patients and providers at an appropriate time. For multimorbidity, integration of CDSSs with telemonitoring could be helpful for patients who need close monitoring of their health status but do not have an indication for hospital admission, as they can be monitored at home and can keep a sense of contact with their providers [39]; this includes patients with cardiovascular disease [40], allergic diseases [41], and COVID-19 [42].

For consultation and case management, integrating CDSSs with strategies to shift tasks from more specialized to less specialized health providers could reduce the burden of management for multimorbidity and increase the impact of health care [43]. CDSSs can reduce the workload of medical staff by automating responses to common patient queries and increasing the potential of nonprofessional providers to manage patient problems. This is relevant for managing multimorbidity, as evidence-based and real-time data and algorithms can improve decision-making for community health workers and primary care providers, helping them to make more accurate decisions across the multitude of situations they encounter [44]. Online platforms allow them to consult with specialists when the situation needs expert opinion and management. However, when incorporating CDSSs into primary care and the community, it is important to develop a feasible and acceptable intervention for frontline health workers. Digital tool training and internet connectivity in the areas where the CDSS will be used must also be provided [45].

The review identified several gaps in evidence when examining the incorporation of CDSSs into telemedicine to manage multimorbidity. First, there was a small number of clinical trials and a lack of diversity in chronic conditions. This review summarized the interventions applied for people with hypertension, diabetes, polypharmacy, and atrial fibrillation, but these 4 conditions are likely inadequate to represent all major chronic diseases commonly found in patients with multimorbidity. A study conducted in Cambodia, Myanmar, Thailand, and Vietnam revealed that the 3 most common chronic conditions with comorbidities were hypertension (37.4%), depression (34.4%), and digestive diseases (32%) [46]. Other evidence shows that depression, diabetes, cancer, and cardiovascular disease are the most common starting conditions before the accumulation over time of comorbidities and conditions, such as osteoarthritis and dementia [47-49]. It should be kept in mind that mental illness and cancer, diseases often associated with multimorbidity that account for a large proportion of coexisting conditions [50-55], were not found in this review.

Second, there was limited evidence for using CDSSs for screening, whether for early prevention or early diagnosis. This might be due to the inclusion criteria of the studies, which allowed only patients who were already diagnosed with multiple diseases to enroll. However, the previous evidence shows that CDSSs could potentially be used to help identify health issues in people who are not currently experiencing symptoms (ie, early detection and screening), such as dementia [56], cardiovascular disease [57], breast cancer [58,59], and pancreatic cancer [60]. Studies investigating the role of CDSSs in detecting multiple chronic diseases are still in the area of active research and development.

Third, the role of CDSSs for patients with multimorbidity remained relatively underexamined. Most of the feedback (eg, prompts and alerts) was sent to providers rather than to patients. Patients are informed less, and providers are the main decision-makers who decide which further steps of treatment should be taken. Low patient engagement makes patient-centered services difficult and could lead to negative consequences, such as poorer health outcomes, decreased adherence to the treatment, and lack of trust in health care providers [61]. Therefore, the ICT system should provide more opportunities for patients to be involved, such as by regularly giving them feedback, informing them about their health status, and delegating some decision-making responsibility to them.

There is a lack of evidence on how telemedicine and CDSS interventions can be used in resource-limited areas. Articles in this review were mostly conducted in high-income countries. While it has been claimed that telemedicine can eliminate geographic barriers and improve access to care, several challenges have been identified as limiting patient access to interventions, including technology access, digital literacy, financial support, regulations on telemedicine use, socioeconomic status, and sociopolitical situations [62,63].

Moreover, the effectiveness of interventions in this review was examined mainly by measuring disease-specific biomarkers, patient behavioral change, and patient care processes in health care facilities. However, as previously mentioned, one major limitation in drawing inferences on effectiveness was the limited number of conditions being assessed. Furthermore, other measures of effectiveness, important for multimorbidity, have not been explored, such as patient-reported outcomes, care coordination, and cost-effectiveness. For multimorbidity, patient-reported outcome measures are an essential tool to assess patients’ perspectives on their health status and the impact of interventions. They provide information for achieving health system goals and evaluating patients’ quality of care [64]. Measuring care coordination, which includes establishing responsibility, communication, goal setting, care plan development, and monitoring, can potentially be useful [65]. And cost-effectiveness studies are likely needed to help analyze the price of inputs for one unit gained of health outcomes of the intervention. This analytic tool compares the cost of the intervention with the cost of alternatives to achieve the same goal [66,67]. This is highly relevant for ICT interventions where infrastructure and workforce to support delivery can still be limited in developing countries [68].

It should also be noted that the literature suggests that there are multiple barriers to adopting CDSSs and telemedicine [69]. Software development requires huge budgets and a large number of work hours; thus, the intervention’s cost and benefits need to be carefully estimated before investment, but as reflected in the review, data are limited for people with multimorbidity. Another barrier to implementation is acceptance by health care providers. On the one hand, CDSSs and telemedicine provide useful information to promote health, assist treatment, and improve patient care. On the other hand, it has been claimed by physicians that it causes significant delays in their daily routine. This finding was related to evidence that health care professionals will accept the intervention after considering how it affects patient disease management and workflows [70]. Some experts felt that this technology could not replace their skills and experience in disease diagnosis and management and that the ability of CDSSs to work across different care settings was still questionable [69]. Hence, making patients and health care workers satisfied with the technology calls for system designs that are both feasible and usable. A process evaluation of how to reduce time used for CDSSs and telemedicine in routine daily care workflows and how to make providers adopt the program is likely needed.

### Strengths and Limitations

The novelty of this review is the attempt to summarize how CDSSs have been integrated into telemedicine services for patients with multimorbidity in primary care. However, there are some limitations. Telemedicine, CDSSs, and multimorbidity are broad concepts. Despite a comprehensive search strategy, some relevant literature may have been missed. A past review exploring the role of CDSSs and telemedicine in critical care [71] included only 2 studies, while a review examining the role of CDSSs and telemedicine in prehospital emergency care [72] included 7 studies. Our review was limited to 4 databases (PubMed, Embase, CINAHL, and Cochrane) and to literature published in English. Thus, non-English articles and articles that were published in other databases may have been missed. All the included studies were conducted in high-income countries with variations in design, sample size, study protocol, and outcomes. This makes it difficult to compare effectiveness across studies and make a clear summary or conclusion. Moreover, digital health is new in the medical research field, and the number of studies is still limited; ongoing research might have been missed.

### Conclusion

There is a role for telemedicine and CDSSs in supporting patients with multimorbidity. CDSSs can likely be integrated into telehealth services to improve the quality and accessibility of care, resulting in a reduction in the burden of disease. However, various aspects of such interventions need to be explored, such as ways to expand the tasks included in CDSSs and the role of patient involvement, and interventions for a greater variety of conditions should be included. Additional assessments of the effectiveness of the intervention are required, including patient-reported outcomes, care coordination, and cost-effectiveness. Moreover, as the interventions have heterogeneous purposes and situational contexts, the feasibility and usability of the systems from the points of view of both health care providers and patients should be investigated in order to develop an ICT system that meets the needs of the local context.

## Appendix

Multimedia Appendix 1 Search terms in different databases.

Multimedia Appendix 2 Search terms for extended search.

Multimedia Appendix 3 The functions of telemedicine.

Multimedia Appendix 4 Types of clinical decision support system task in telemedicine.

Multimedia Appendix 5 Summary of types of intervention outcome.

## Abbreviations

ADE: adverse drug event
CDSS: clinical decision support system
EHR: electronic health record
HbA_1c_: hemoglobin A_1c_
ICT: information and communication technology
PRISMA: Preferred Reporting Items for Scoping Reviews
RCT: randomized control trial
