# Supplementary material for: The Integration of Clinical Decision Support Systems Into Telemedicine for Patients With Multimorbidity in Primary Care Settings: Scoping Review
Source: J Med Internet Res. 2023 Jun 28;25:e45944. doi: 10.2196/45944 (PMC10365574; doi:10.2196/45944)
Supplement: Multimedia Appendix 1 [file jmir_v25i1e45944_app1.docx]

**Appendix 1** Search terms in different databases

*Pubmed*

| P | Step | Words | Found |
| --- | --- | --- | --- |
|  | #1 | Polypharmacy [MeSH] | 11562 |
|  | #2 | multiple chronic conditions [MeSH] | 12037 |
|  | #3 | multiple pharmacotherapy[Title/Abstract] | 8 |
|  | #4 | Multiple drug treatment [Title/Abstract] | 81 |
|  | #5 | multi-drug therapy[Title/Abstract] | 371 |
|  | #6 | polypragmasia[Title/Abstract] | 35 |
|  | #7 | multidrug therapy[Title/Abstract] | 1309 |
|  | #8 | multiple chronic illnesses [Title/Abstract] | 119 |
|  | #9 | concurrent chronic [Title/Abstract] | 323 |
|  | #10 | simultaneous chronic[Title/Abstract] | 52 |
|  | #11 | multimorbidity[Title/Abstract] | 6024 |
|  | #12 | #1 OR #2 OR #3 OR #4 OR #5 OR #6 OR #7 OR #8 OR #9 OR #10 OR #11 | 30119 |
| I | Step | Words | Found |
|  | #13 | clinical decision support system [MeSH] | 25085 |
|  | #14 | cds[Title/Abstract] | 15598 |
|  | #15 | clinical decision support system* [Title/Abstract] | 2568 |
|  | #16 | clinician decision support system* [Title/Abstract] | 1 |
|  | #17 | #13 OR #14 OR #15 OR #16 | 39861 |
| Combine | #18 | #12 AND #17 | 290 |
|  | #19 | #18 AND Publication data Filters applied: from 2015-2021 | 211 |

*EMBASE*

('polypharmacy'/exp OR polypharmacy OR 'multi-drug therapy' OR 'multidrug therapy' OR 'multiple drug therapy' OR 'multiple drug treatment' OR 'multiple pharmacotherapy' OR 'pharmacotherapy, multiple' OR 'pharmacy, poly' OR 'poly pharmacy' OR 'polypharmacy' OR 'polypragmasia' OR 'polypragmasy' OR 'multiple chronic conditions'/exp OR 'concurrent chronic conditions' OR 'concurrent chronic diseases' OR 'concurrent chronic disorders' OR 'concurrent chronic health conditions' OR 'concurrent chronic illnesses' OR 'concurrent chronic medical conditions' OR 'multimorbidity' OR 'multiple chronic condition' OR 'multiple chronic conditions' OR 'multiple chronic diseases' OR 'multiple chronic disorders' OR 'multiple chronic health conditions' OR 'multiple chronic illnesses' OR 'multiple chronic medical conditions' OR 'simultaneous chronic illnesses' OR 'simultaneous chronic medical conditions’)

AND

('clinical decision support system'/exp OR 'cds system (clinical decision support system)' OR 'clinical decision support (cds) system' OR 'clinical decision support system' OR 'clinical decision support systems' OR 'decision support systems, clinical’)

Total 111 articles

Pub data filter applied: 103 titles

*Cochrane*

| P | Step | Words | Found |
| --- | --- | --- | --- |
|  | #1 | Polypharmacy [MeSH] | 227 |
|  | #2 | multiple chronic conditions [MeSH] | 38 |
|  | #3 | multiple pharmacotherapy[Title/Abstract/Keyword] | 344 |
|  | #4 | Multiple drug treatment [Title/Abstract/Keyword] | 29172 |
|  | #5 | multi-drug therapy[Title/Abstract/Keyword] | 342 |
|  | #6 | polypragmasia[Title/Abstract/Keyword] | 2 |
|  | #7 | multidrug therapy[Title/Abstract/Keyword] | 1583 |
|  | #8 | multiple chronic illnesses [Title/Abstract/Keyword] | 165 |
|  | #9 | concurrent chronic [Title/Abstract/Keyword] | 1164 |
|  | #10 | simultaneous chronic[Title/Abstract/Keyword] | 623 |
|  | #11 | multimorbidity[Title/Abstract/Keyword] | 454 |
|  | #12 | #1 OR #2 OR #3 OR #4 OR #5 OR #6 OR #7 OR #8 OR #9 OR #10 OR #11 | 33148 |
| I | Step | Words | Found |
|  | #13 | clinical decision support system/Information system [MeSH] | 2409 |
|  | #14 | cds[Title/Abstract/Keyword] | 598 |
|  | #15 | clinical decision support system* [Title/Abstract/Keyword] | 2571 |
|  | #16 | clinician decision support system* [Title/Abstract/Keyword] | 257 |
|  | #17 | #13 OR #14 OR #15 OR #16 | 5000 |
| Combine | #18 | #12 AND #17 | 149 |
|  | #19 | #18 AND Publication data Filters applied: from 2015-2021 | 105 |

*CINAHL*

| P | Step | Words | Found |
| --- | --- | --- | --- |
|  | #1 | Polypharmacy [MeSH] | 4955 |
|  | #2 | multiple chronic conditions [MeSH] | 2222 |
|  | #3 | multiple pharmacotherapy [TX All text] | 157 |
|  | #4 | Multiple drug treatment [TX All text] | 960 |
|  | #5 | multi-drug therapy[TX All text] | 240 |
|  | #6 | Polypragmasia [TX All text] | 6 |
|  | #7 | multidrug therapy [TX All text] | 1939 |
|  | #8 | multiple chronic illnesses [TX All text] | 1259 |
|  | #9 | concurrent chronic [TX All text] | 802 |
|  | #10 | simultaneous chronic[TX All text] | 192 |
|  | #11 | multimorbidity[TX All text] | 5021 |
|  | #12 | #1 OR #2 OR #3 OR #4 OR #5 OR #6 OR #7 OR #8 OR #9 OR #10 OR #11 | 5021 |
| I | Step | Words | Found |
|  | #13 | clinical decision support system [MeSH] | 5772 |
|  | #14 | cds[TX All text] | 7697 |
|  | #15 | clinical decision support system* [TX All text] | 60940 |
|  | #16 | clinician decision support system*[TX All text] | 14 |
|  | #17 | #13 OR #14 OR #15 OR #16 | 68131 |
| Combine | #18 | #12 AND #17 | 553 |
|  | #19 | #18 AND Publication data Filters applied: from 2015-2021 | 410 |
