# Supplementary material for: The Integration of Clinical Decision Support Systems Into Telemedicine for Patients With Multimorbidity in Primary Care Settings: Scoping Review
Source: J Med Internet Res. 2023 Jun 28;25:e45944. doi: 10.2196/45944 (PMC10365574; doi:10.2196/45944)
Supplement: Multimedia Appendix 2 [file jmir_v25i1e45944_app2.docx]

**Appendix 2** search terms for extended search

| P | Steps | Words | Found |
| --- | --- | --- | --- |
|  | #1 Diabetes | "Diabetes Mellitus, Type 2"[Mesh] OR "Diabetes Mellitus, Type 1"[Mesh] | 214,840 |
|  | #2 Cardiovascular disease | "Cardiovascular Diseases"[Mesh] OR "Myocardial Ischemia"[Mesh] OR "Stroke"[Mesh] OR "Intracranial Embolism and Thrombosis"[Mesh] OR "Intracranial Hemorrhages"[Mesh] | 2,556,447 |
|  | #3 Hypertension | "hypertension"[MeSH] OR "high blood pressure"[All Fields] | 308,717 |
|  | #4 Dyslipidemia | "hyperlipidemias"[MeSH] OR "hypertriglyceridemia"[MeSH] OR "hypercholesterolemia"[MeSH] OR "dyslipidemias"[MeSH] | 83,455 |
|  | #5 Heart failure | "Heart Failure"[Mesh] | 133,430 |
| Combine | #1 OR #2 OR #3 OR #4 OR #5 | #6 | 2,770,994 |
| I | #7 CDSS | "Decision Support Systems, Clinical"[Mesh] | 8,818 |
| Combine | #6 AND #7 |  | 1,021 |
|  | 5 year filter |  | 284 |
