# Supplementary material for: The Integration of Clinical Decision Support Systems Into Telemedicine for Patients With Multimorbidity in Primary Care Settings: Scoping Review
Source: J Med Internet Res. 2023 Jun 28;25:e45944. doi: 10.2196/45944 (PMC10365574; doi:10.2196/45944)
Supplement: Multimedia Appendix 4 [file jmir_v25i1e45944_app4.docx]

**Appendix 4:** The types of CDSS task in telemedicine

|  | Prevention | Diagnosis | Guideline or Pathway | Medication | Patient education | Self-management |
| --- | --- | --- | --- | --- | --- | --- |
| Fried T. 2017 |  |  |  | √ |  |  |
| Marcolino 2021 |  | √ | √ |  |  |  |
| McDonald 2019 |  |  |  | √ | √ |  |
| Peleg 2017 |  |  | √ |  |  | √ |
| Schiff 2019 |  |  |  | √ |  |  |
| Willis 2020 |  |  | √ | √ | √ | √ |
| Prabhakaran 2018 |  |  | √ |  | √ | √ |
