# Supplementary material for: The Integration of Clinical Decision Support Systems Into Telemedicine for Patients With Multimorbidity in Primary Care Settings: Scoping Review
Source: J Med Internet Res. 2023 Jun 28;25:e45944. doi: 10.2196/45944 (PMC10365574; doi:10.2196/45944)
Supplement: Multimedia Appendix 5 [file jmir_v25i1e45944_app5.docx]

**Appendix 5:** The summary of types of intervention outcomes

|  | Disease control | Medication management | Patient self-management | Patient care | Doctor-patient communication | Feasibility and satisfaction of intervention |
| --- | --- | --- | --- | --- | --- | --- |
| Fried T. 2017 |  | + |  |  | + |  |
| Marcolino 2021 |  |  |  |  |  | + |
| McDonald 2019 |  | + |  |  |  |  |
| Peleg 2017 |  |  | + | + |  |  |
| Schiff 2019 |  | + |  |  |  |  |
| Willis 2020 |  |  | + |  |  |  |
| Prabhakaran 2018 | 0 |  | 0 |  |  |  |

+ positive results, 0 not statistically significant
